# The *Pro12Ala* polymorphism in the *PPAR-γ2* gene is not associated to obesity and type 2 diabetes mellitus in a Cameroonian population

**DOI:** 10.1186/s40608-016-0104-6

**Published:** 2016-05-12

**Authors:** Edith Pascale M. Mato, Priscille Eunice Pokam-Fosso, Barbara Atogho-Tiedeu, Jean Jacques N. Noubiap, Marie-Solange Evehe, Rosine Djokam-Dadjeu, Olivier Sontsa Donfack, Elvis Ndonwi Ngwa, Magellan Guewo-Fokeng, Wilfred F. Mbacham, Eugene Sobngwi, Jean Claude Mbanya

**Affiliations:** Department of Biochemistry, Faculty of Science, University of Yaoundé I, Yaoundé, Cameroon; Laboratory for Molecular Medicine and Metabolism, Biotechnology Center, University of Yaoundé I, Yaoundé, Cameroon; Department of Medicine, Groote Schuur Hospital and University of Cape Town, Cape Town, South Africa; Medical Diagnostic Center, Yaoundé, Cameroon; Laboratory for Public Health Research and Biotechnologies, Biotechnology Center, University of Yaoundé I, Yaoundé, Cameroon; Department of Internal Medicine and Specialties, Faculty of Medicine and Biomedical Sciences, University of Yaoundé I, Yaoundé, Cameroon; National Obesity Center, Yaoundé Central Hospital and Faculty of Medicine and Biomedical Sciences, University of Yaoundé 1, Yaoundé, Cameroon

**Keywords:** Obesity, Type 2 diabetes mellitus, Genetic association, *PPAR-γ2*, *PPAR gamma2*, Pro12Ala

## Abstract

**Background:**

Peroxisome proliferator-activated receptor gamma 2 (*PPAR-γ2*) is a transcription factor with a key role in adipocyte differentiation, lipid storage and glucose homeostasis. The Ala allele of the common Pro12Ala polymorphism in the isoform *PPAR-γ2* is at the center of many controversies because in some populations, it has been observed to be associated with T2DM or obesity but, not in others. The aim of this study was to investigate the association of Pro12Ala polymorphism in the *PPAR-γ2* gene with susceptibility to obesity or T2DM in a Cameroonian population.

**Methods:**

This case-control study included 62 obese, 60 T2DM patients and 120 controls (60 non obese and 60 patients without T2DM), all unrelated and of Cameroonian origin. *PPAR-γ2* was examined by genotyping for *Pro12Ala* using the Restriction Fragment Length Polymorphism - Polymerase Chain Reaction (PCR - RFLP).

**Results:**

A portion of the 270 base pair bands of the *PPAR-γ2* gene was successfully amplified. The *Ala12* variant was totally absent from the study population, all participants being homozygote *Pro/Pro*.

**Conclusion:**

*PPAR-γ2 Pro12Ala* gene polymorphism may not be associated with obesity and T2DM. These results suggest that, *PPAR-γ2* is unlikely a major gene for obesity or T2DM in the study population.

## Background

Type 2 diabetes mellitus and obesity are a major global health problem. The prevalence of obesity and T2DM is increasing dramatically and WHO reports that more than 500 million people are obese [1] and 387 million have diabetes [2]. T2DM is a metabolic disease characterized by hyperglycemia that can occur as a result of impaired insulin secretion, insulin resistance in peripheral tissues and increased hepatic glucose output [3], while obesity occurs as the result of chronic energy imbalance involving physical inactivity, excessive energy intake, depression, sleep disorders and low socioeconomic status [4]. Obesity and T2DM are called “multifactorial” or “complex” diseases because, they depend on multiple genetic factors as well as environment.

Some genes are thought to be involved in the development of T2DM and obesity amongst which, the peroxisome proliferator activated receptor gamma *(PPAR-γ)* gene. *PPAR-γ* is a member of a subfamily of the nuclear receptor superfamily of a ligand activated transcription factors (PPAR) that consists of 3 subtypes: PPAR-*α*, PPAR-*β* and PPAR-*γ. PPARγ2* which results from mRNA alternative splicing of *PPAR-γ* is expressed predominantly in the adipose tissue. It has been shown that *PPAR-γ* has an important role in adipocyte differentiation and that it regulates lipid metabolism and sensitivity to insulin [5]. A CCA-GCA mutation leading to the substitution of proline by alanine on codon 12 of exon B of the *PPAR-γ2* has been described. This mutation was first identified by Schuldiner’s group in 1997 with different ethnic population showing various allelic frequencies that is probably modulated by environmental and other genetics factors. Caucasians have the highest frequency (12 %), followed by Mexican Americans (10 %), West Samoans (8 %), African Americans (3 %), while Chinese have the lowest (1 %) [6]. In Africans and Asians, frequency of Ala 12 is low (1–3 %). The *Ala* isoform may lead to a less efficient stimulation of the PPAR-γ target genes and predisposes to lower levels of adipose tissue mass accumulation which in turn may be responsible for improved insulin sensitivity [7]. The large and increasing burden of non communicable diseases and the potential for modifying risk through adequate treatments and lifestyle alterations make the identification of methods for early detection of persons at greater risk an important public health challenge. Knowledge of genetic polymorphisms that allow accurate quantification of T2DM and obesity risk will allow the development of complex models with diagnostic and prognostic. Many studies clearly show heterogeneous effects of this polymorphism on predicting susceptibility to the risk of T2DM and obesity in various populations [8–10]. This preliminary study aimed to investigate the association of *Pro12Ala* with T2DM and obesity in a Cameroonian population.

## Methods

### Study population

This is a case-control study involving 62 obese adults and 60 non-obese controls, and 60 T2DM patients and 60 non-diabetic healthy controls of Cameroonian origin, aged 20 years old and above. Obese and T2DM patients were recruited from the outpatient clinic of the National Obesity Center of the Yaoundé Central Hospital, and non-obese controls from the general population. For all participants, data was collected on the sex and age; we measured height, waist and hip circumference to the nearest 0.5 cm, and weight in light clothes to the nearest 0.1 kg, and we then calculated the body mass index (BMI) as weight in kg/height^2^ in m^2^, and the waist-to-hip ratio. Obesity was defined as a BMI ≥30 kg/m^2^. We measured the resting blood pressures using standardized procedures with an automatic sphygmomanometer Omron HEM-705 CP (Omron Corporation, Tokyo, Japan).

### Biochemical assays

Fasting plasma glucose (glucose oxidase–peroxidase method), serum cholesterol (cholesterol oxidase phenol4-amino antipyrene peroxidase method), serum triglycerides (glycerol phosphatase oxidase − phenol4-amino antipyrene peroxidase method), and high-density lipoprotein (HDL)-cholesterol (cholesterol oxidase phenol4-amino antipyrene peroxidase method) were measured on a spectrophotometer (UV Mini 1240) using Chronolab kits (Chronolab Systems, Barcelona, Spain). Low-density lipoprotein (LDL)-cholesterol was calculated using the Friedwald’s formula [11].

### DNA extraction and molecular genotyping

Genomic DNA was isolated from dried blood spots on Whatmann filter paper by the chelex method [12]. The Pro12Ala polymorphism of the *PPARγ2* gene was analysed using the Polymerase Chain Reaction-Restriction Fragment Length Polymorphism (PCR-RFLP) method. Exon B was amplified with a T3 Thermocycler (BIOMETRA) using the forward primer: 5′-GCCAATTCAAGCCCAGTC-3′ and the reverse primer: 5′-GATATGTTTGCAGACAGTGTATC-3′ (SIGMA-ALDRICH). Each PCR was performed in a final volume of 15 μl containing 100 ng of DNA extract, 5 pmol of each primer, 100 μmol/L of each deoxynucleotide triphosphate (dNTP), 0.5 U of Hot Star Taq DNA polymerase (QIAGEN), 1.5 μl of PCR buffer with 1 mmol/L of MgCl_2_ and 7.8 μl of nuclease free water. The reaction was carried out under the following conditions: predenaturation at 95 °C for 15 min, followed by 30 cycles of denaturation at 95 °C for 1 min, annealing at 56 °C for 1 min and elongation at 72 °C for 1 min, and a final extension at 72 °C for 10 min. Electrophoresis was conducted to confirm the 270 bp PCR products. Six microliters of positive PCR products were then digested at 37 °C overnight with 0.2 μl of the B*st* UI restriction endonuclease using the New England Buffer 4 recommended by the manufacturer (NEW ENGLAND BIOLABS, UK). Digestion products were analysed by electrophoresis using a 3 % agarose gel. In order to verify whether our samples contained an enzyme inhibitor, we carried out a mix-in experiment using the T*sp* 5091 restriction endonuclease to digest the mixture containing Pro12Ala amplicons and rs 12255372 amplicons with a restriction site for the T*sp* 5091 enzyme.

### Statistical analysis

Statistical analysis was performed using IBM SPSS for Windows, version 20.0 (IBM Corp., Armonk New York, USA). Quantitative variables were expressed as mean (Standard deviation (SD). The clinical and laboratory characteristics of cases and controls were compared using chi-square tests. The continuous variables have been studied by ANOVA T test, Mann Whitney U test. A *p* value < 0.05 was considered statistically significant.

## Results

The biochemical and anthropometric results for the study population are shown in Tables 1 and 2. In the group of obese status (Table 1), fasting blood glucose was significantly higher in obese subjects (*p* = 0.027), as well as systolic blood pressure (*p* < 0.001) and diastolic blood pressure (*p* < 0.001). Considering lipid profile, only total cholesterol levels were significantly higher in obese subjects compared to non-obese ones (*p* = 0.04). While, in the group of T2DM status (Table 2), the diastolic blood pressure (*p* =0.034), total Cholesterol (*p* < 0.001), LDL - Cholesterol (*p* < 0.001) and the atherogenic index (*p* < 0.001) were significantly higher in non T2DM patients compared to the T2DM patients. The genotype analysis revealed that a portion of the 270pb band of the *PPAR-γ2* gene.*Ala12* variant was totally absent from the study population, all participants being homozygote *Pro/Pro*.

**Table 1:** Characteristics of obese patients and controls

Parameters	Cases (*n* = 62)	Controls (*n* = 60)	*p* value
Age (years)	43 (11)	34 (12)	<0.001
BMI (Kg/m^2^)	34.22 (4.62)	22.10 (1.70)	<0.001
WHR	0.86 (0.07)	0.81 (0.06)	<0.001
FPG (mg/dL)	96.81 (11.74)	92.03 (11.08)	0.027
SBP (mmHg)	131.13 (20.05)	116.98 (16.45)	<0.001
DBP (mmHg)	80.53 (10.90)	70.77 (10.97)	<0.001
T-C (mg/L)	190.5 (35.89)	180.68 (26.85)	0.040
TG (mg/dL)	135.79 (19.47)	131.81 (18.26)	0.247
HDL-C (mg/dL)	49.02 (7.89)	47.89 (5.37)	0.360
LDL-C (mg/dL)	114.84 (33.29)	106.46 (25.02)	0.119
AI	3.93 (0.68)	3.80 (0.62)	0.293

Data are expressed as mean (standard deviation)

*FPG* Fasting Plasma Glucose, *SBP* Systolic Blood Pressure, *DBP* Diastolic Blood Pressure, *HDL-C* high density lipoprotein cholesterol, *LDL-C* low density lipoprotein cholesterol, *TG* triglycerides, *AI* Atherogenic index = total cholesterol/HDL-cholesterol

**Table 2:** Characteristics of the study population (T2DM status)

Parameters	(*n* = 60)	Controls (*n* = 60)	*p* value
Age (years)	60 (20)	50 (12)	<0.001
BMI (Kg/m^2^)	29.08 (5.74)	29 (4.76)	0.602
WHR	0.95 (0.08)	0.87 (0.07)	<0.001
FPG (g/L)	1.78 (0.77)	0.92 (0.09)	<0.001
SBP (mmHg)	137 (24)	138 (25)	0.775
DBP (mmHg)	79 (12)	85 (14)	0.034
T-C (g/L)	1.65 (0.23)	1.97 (0.39)	<0.001
TG (g/L)	1.42 (0.29)	1.49 (0.27)	0.171
HDL-C (g/L)	0.49 (0.10)	0.51 (0.6)	0.274
LDL-C (g/L)	0.88 (0.21)	1.16 (0.36)	<0.001
AI	2.31 (0.68)	1.86 (0.56)	<0.00 1

Data are expressed as mean (standard deviation)

*FPG* Fasting Plasma Glucose, *SBP* Systolic Blood Pressure, *DBP* Diastolic Blood Pressure, *HDL-C* high density lipoprotein cholesterol, *LDL-C* low density lipoprotein cholesterol, *TG* triglycerides, *AI* Atherogenic index = total cholesterol/HDL-cholesterol

## Discussion

Africa is experiencing a surge on obesity prevalence and related morbidity and mortality within the context of one of the most rapid epidemiological transition in the world history [1, 2]. Obesity and T2DM are caused by combined effect of many factors that could be environmental, psychosocial and genetic. Although the epidemic is attributable to the trend of decreased physical activity and increase caloric intake, these external factors are playing out on a genetic background to determine body mass and susceptibility to obesity-related disease [4]. From the point of view of Evolutionary Genetics, it has been hypothesized that there are some “thrifty genes” that have been fixed in our genome for thousands of years to prevent hunger, cold and calamities which hinder feeding [13]. On the contrary, *PPAR* γ2 is an “unthrifty” gene which has been shown to play an important role in adipocyte differentiation, regulation of lipid metabolism and sensitivity to insulin [7, 14, 15]. It has a protective effect against type 2 diabetes mellitus, and is associated to obesity [14–16]. Data on the genetic epidemiology of *PPAR* γ2 are very scare in populations of African ancestry. This study aimed to investigate the frequency of the proline to alanine substitution in the human *PPAR* γ2 gene and its association with obesity and T2DM in a Cameroonian population. We found that the *Ala* allele was completely absent in our study population, and the *Pro* allele was fixed. The frequencies of the *Ala* vary widely across ethnic populations. A typical black africans population (Berba, African Bantu) shows no Ala12 allele [17], whereas Ethiopian African population (Amhara) [17], African American population (African ancestry on Southwest USA) [18] and Oriental populations (Japanese, Singapore Chinese, Han Chinese and Koreans) exhibit a small Ala12 frequency [19–22]. Typical Far East Asian populations and North African Caucasoids (Tunisians) and Middle East Caucasoids (Qataris) show also a Ala12 low frequency [23, 24]. North Indian Sikhs have a high Ala12 mutation frequency [25], as well as Europeans and Amerindians [13, 26, 27]. Our findings are therefore consistent with data from the literature which show that; overall, black africans tend to show low or null Ala12 allele frequencies, while most other populations have a significant frequency, particularly European Caucasoids. These genetic epidemiologic patterns may be explained by the fact that the Pro12 allele seems to be the wild allele which must have occurred before modern humans left Africa [14], while the Ala 12 allele must have occurred in Caucasoids later on [14]. The variability in Ala 12 frequencies in Caucasians and Caucasoids may have been due to evolutionary fitness forces pertaining to feeding and metabolic adaptation.

## Conclusion

This study suggests that *PPAR-γ2 Pro12Ala* mutation is not associated with the development of obesity and T2DM in the Cameroonian population. Our findings are consistent with existing data which show no Ala12 allele in typical black africans populations.

## Ethics statement

The study was granted approval by the National Ethical Review Board of the Cameroon Ministry of Public Health. Written informed consent was obtained from all the participants. The study was conducted in accordance with the Helsinki Declaration.

## Availability of data and materials

Data will be made available by the corresponding author upon request.

## Abbreviations

ANOVA: analysis of variance
DNA: deoxyribonucleic acid
PCR: polymerase chain reaction
*PPAR-γ2*: peroxisome proliferator-activated receptor gamma 2
T2DM: type 2 diabetes mellitus
WHO: World Health Organization
